# Did Smokefree Legislation in England Reduce Exposure to Secondhand Smoke among Nonsmoking Adults? Cotinine Analysis from the Health Survey for England

**DOI:** 10.1289/ehp.1103680

**Published:** 2011-12-12

**Authors:** Michelle Sims, Jennifer S. Mindell, Martin J. Jarvis, Colin Feyerabend, Heather Wardle, Anna Gilmore

**Affiliations:** 1 Department for Health, and the UK Centre for Tobacco Control Studies, University of Bath, Claverton Down, Bath, United Kingdom; 2 Department of Epidemiology and Public Health, University College London, London, United Kingdom; 3 ABS (Advanced Bioanalytical Service) Laboratories, BioPark, Welwyn Garden City, Hertfordshire, United Kingdom; 4 National Centre for Social Research, London, United Kingdom

**Keywords:** cotinine, environmental tobacco smoke, evaluation, nonsmokers, passive smoke, secondhand smoke, smokefree legislation, smoking ban

## Abstract

**Background:**

On 1 July 2007, smokefree legislation was implemented in England, which made virtually all enclosed public places and workplaces smokefree.

**Objectives:**

We examined trends in and predictors of secondhand smoke exposure among nonsmoking adults to determine whether exposure changed after the introduction of smokefree legislation and whether these changes varied by socioeconomic status (SES) and by household smoking status.

**Methods:**

We analyzed salivary cotinine data from the Health Survey for England that were collected in 7 of 11 annual surveys undertaken between 1998 and 2008. We conducted multivariate regression analyses to examine secondhand smoke exposure as measured by the proportion of nonsmokers with undetectable levels of cotinine and by geometric mean cotinine.

**Results:**

Secondhand smoke exposure was higher among those exposed at home and among lower-SES groups. Exposure declined markedly from 1998 to 2008 (the proportion of participants with undetectable cotinine was 2.9 times higher in the last 6 months of 2008 compared with the first 6 months of 1998 and geometric mean cotinine declined by 80%). We observed a significant fall in exposure after legislation was introduced—the odds of having undetectable cotinine were 1.5 times higher [95% confidence interval (CI): 1.3, 1.8] and geometric mean cotinine fell by 27% (95% CI: 17%, 36%) after adjusting for the prelegislative trend and potential confounders. Significant reductions were not, however, seen in those living in lower-social class households or homes where smoking occurs inside on most days.

**Conclusions:**

We found that the impact of England’s smokefree legislation on secondhand smoke exposure was above and beyond the underlying long-term decline in secondhand smoke exposure and demonstrates the positive effect of the legislation. Nevertheless, some population subgroups appear not to have benefitted significantly from the legislation. This finding suggests that these groups should receive more support to reduce their exposure.

There is now a large body of evidence documenting the adverse effects of secondhand smoke (SHS, also called environmental tobacco smoke) exposure among nonsmoking adults. These effects mirror those associated with active smoking, with SHS exposure now causally linked with coronary heart disease, lung and nasal sinus cancer and associated with an increased risk of other cancers, stroke, chronic respiratory symptoms and adverse pregnancy outcomes (California Environmental Protection Agency 2005). Although most reported relative risks are generally low, large numbers of nonsmokers are at risk because exposure has historically been ubiquitous, occurring in the home, at work, in enclosed public places and other social settings, and the population harm arising from exposure to SHS is therefore significant. Measures to reduce exposure to SHS, including legislation prohibiting smoking in public and work places, are therefore expected to result in health benefits (U.S. Department of Health and Human Services 2006).

Comprehensive smoking bans have now been implemented in several jurisdictions worldwide. Evaluations of the legislation document substantial improvements in air quality in public venues and reductions in SHS exposure and related respiratory and sensory symptoms in hospitality workers and patrons [International Agency for Research on Cancer (IARC) 2009]. Evidence of health benefits, most notably reductions in hospital admissions for coronary events, in the general population is also accumulating (Barone-Adesi et al. 2011; Juster et al. 2007; Naiman et al. 2010; Sims et al. 2010a). Such evaluations presuppose that legislation reduces SHS exposure among the general population, yet only a few studies, one in Scotland and two in the United States, have yet examined this using a specific biomarker of tobacco smoke exposure (Adda and Cornaglia 2010; Bauer et al. 2007; Haw and Gruer 2007). The two that examined implementation of specific legislation found significant reductions in exposure among nonsmoking adults, although neither was able to account for underlying secular declines in exposure (Bauer et al. 2007; Haw and Gruer 2007). The third was conducted by Adda and Cornaglia (2010) who used regression models to examine whether cotinine levels varied with the extent of smokefree legislation in U.S. states; they found no significant impact among nonsmoking adults. That study, however, has been critiqued because the investigators were unable to fully account for whether individuals lived in municipalities or counties that were smokefree (IARC 2009).

In this study, we aimed to determine whether the introduction of smokefree legislation in England on 1 July 2007 led to changes in SHS exposure among nonsmoking adults (Department of Health 2008). Such exposure has been found, both in the United States and in England, to vary by the extent of smoking in the home and socioeconomic status (SES) (Jarvis et al. 2001; Pirkle et al. 2006; Royal College of Physicians 2005). Therefore, we explored whether these inequalities in exposure changed after legislation was introduced. In addition, we report on the trend in SHS exposure among the general population in England from 1998 to 2008, updating an earlier study (Royal College of Physicians 2005). Finally, because previous analyses in England have focused only on nonsmoking adults living with a partner who smokes (Jarvis et al. 2001) and on children (Sims et al. 2010b), we explored other factors related to SHS exposure in detail.

## Methods

*Data.* We obtained 11 years of data (1998 to 2008 inclusive) from the Health Survey for England, an annual, representative, cross-sectional survey of individuals living in private households in England (Department of Health 2010; NHS Information Centre 2010a). The survey design ensures that the population sampled in each quarter of the year is nationally representative. Data collection involves an interviewer visit, in which all adults ≥ 16 years of age and up to two children in each household are eligible to be interviewed, followed by a nurse visit. In 7 of 11 annual surveys undertaken between 1998 and 2008 [specifically 1998, 2000 (second half of 2000 only—1 July to 31 December), 2001–2003, 2007 and 2008], the nurse collected saliva samples from adults to measure cotinine, a metabolite of nicotine that is considered a reliable marker of tobacco smoke exposure in the previous 72 hr (Benowitz 1996). Salivary cotinine levels were measured using a gas chromatographic method with a lower limit of detection of 0.1 ng/ml (Feyerabend and Russell 1990). In 2008, the methodology was changed to a liquid chromatography tandem mass spectrometry method (LC-MS/MS; Bernert et al. 2009). These two methods produced comparable results for the determination of cotinine (Bernert et al. 2009; Craig et al. 2009). Cotinine levels below this limit are classed as undetectable. We based our analyses on nonsmoking adults, defined as those who self-reported that they did not currently smoke a cigarette, pipe, or cigar and who had a salivary cotinine concentration of < 12 ng/ml (now considered the most accurate cut off for active smoking among adults) (Jarvis et al. 2008).

*Survey design and outcome measures.* We analyzed the data using regression models, with adjustments for the complex survey design involving clustering and stratification; the analyses were performed using the survey package in R (version 2.9.0; The R Foundation for Statisitical Computing, Vienna, Austria). We used weights provided by the Health Survey for England (Craig et al. 2009) to compensate for any nonresponse to the nurse visit. These weights are available from 2003 to 2008. In the 2007 survey, an additional weight was introduced to further adjust for nonparticipation in the saliva sample, and we used this weight when it became available.

Two outcome measures were considered: undetectable cotinine (a binary outcome measure with a value of 1 for those with cotinine below the lower detection limit and 0 for those above the detection limit) and log-transformed cotinine. For the latter, adults with cotinine concentrations below the lower detection limit were assigned a value using regression on order statistics, an imputation method that assigns values to nondetect data based on a probability plot of the detected data (Helsel 2010; Hewett and Ganser 2007). The methods detailed below describe the logistic regression models applied to the binary outcome (undetectable cotinine). These methods were repeated using regression models with normally distributed errors, using the log-transformed cotinine outcome, to model geometric mean cotinine. The geometric mean is considered to be a better summary measure of average cotinine given the skewed distribution of the raw cotinine data. Results in the main text are rounded to two significant figures. All tests were two-sided, and statistical significance was defined as *p* < 0.05.

*Predictors of secondhand smoke exposure.*Univariate regression. Univariate logistic regression analysis was used to examine the relationships between sociodemographic predictors of exposure to SHS and the proportion of nonsmoking adults with undetectable cotinine. Sociodemographic data included age, sex, ethnicity, education of the respondent, social class of the head of household (measured using the British Registrar General’s classification (Office of Population Censuses and Surveys 1991), and whether there was smoking in the home on most days. Smoking in the home was based on the response of the head of household to the question, “Does anyone smoke inside this house/flat on most days?”

Multivariate regression. Multivariate logistic regression was used to estimate the effect of each predictor on the proportion of nonsmoking adults with undetectable cotinine after adjusting for other predictor variables, including long-term trends. To evaluate changes over time, we created a “time” variable (to identify the 6-month period during which saliva samples that were used to determine cotinine levels were collected (6-month periods numbered consecutively from 1, for those with a nurse visit from 1 January to 30 June in the 1998 survey, to 22, for those with a nurse visit from 1 July to 31 December in the 2008 survey). Given the periods of missing cotinine data, quantifying the long-term trend in SHS exposure was difficult. We considered linear, quadratic, and cubic terms for the time variable to model the trend. The cubic term was not significant (*p* > 0.05), but the others were; therefore, we included both linear and quadratic terms in order to model the trend.

*Impacts of legislation.* Overall impact of the smokefree legislation. We examined the impact of smokefree legislation in two ways. First, we included a binary predictor variable in the multivariate model with a value of 1 assigned to adults who had their saliva sample collected after 1 July 2007 and a value of 0 before this date. This model indicated whether there had been an immediate change in the proportion of nonsmoking adults with undetectable cotinine after adjusting for the long-term trend (modeled using both linear and quadratic terms for time) and temporal variation in sociodemographic circumstances.

Second, to avoid making an assumption about the nature of the trend in exposure, we compared the odds of having undetectable cotinine in each 6-month period (as defined by the time variable) with that in the previous 6-month period to assess whether there was a significant increase in the odds of having undetectable cotinine from the first to second half of 2007 and whether the increase was greater than the other 6-month comparisons. Odds and odds ratios (ORs) were calculated using the multivariate logistic regression model, but without the smokefree legislation predictor and with the linear and quadratic terms for time variable replaced by 12 dummy variables, coded in a way that enables a comparison between one 6-month period and the preceding one [see Supplemental Material, pages 2–6 (http://dx.doi.org/10.1289/ehp.1103680)].

Variation in impacts of the smokefree legislation by household smoking status and social class. To assess whether the impacts of smokefree legislation varied by household smoking status, we used a multivariate model, as described above, with the addition of two interaction terms. The first, between household smoking status and the binary indicator of smokefree legislation, assessed whether any change following the introduction of smokefree legislation varied by the household smoking status. The second, between the household smoking status predictor and the linear and quadratic terms for the time variable, adjusts for differences in the long-term trend among these subgroups. This approach was repeated to explore impacts by social class but with household smoking status replaced by social class in the interaction terms.

To validate these findings (and again to avoid making assumptions about the nature of the trend) we applied the logistic regression model in which the odds of having undetectable cotinine in each 6-month period were compared with the odds in the previous 6-month period for each subgroup of adults defined by household smoking status and social class.

## Results

*Determinants of secondhand smoke exposure.* Using undetectable cotinine as the outcome, all predictors were significantly associated with secondhand smoke exposure, except ethnicity (black or Asian vs. white), both in the univariate and multivariate models (Table 1). Having controlled for the other predictors, the odds of having undetectable cotinine increased with age (1.6, 1.8, and 2.2 times that of people 16–29 years of age for those in the 30–44-, 45–59-, and ≥ 60-year age groups, respectively) and decreased with declining SES status with the lowest levels in social class IV and V [29% lower than social class I and II, 95% confidence interval (CI): 21, 35] and in adults with no qualifications (19% lower than those with a higher education qualification, 95% CI: 11, 26). The odds of having undetectable cotinine were 1.2 times higher among women than among men (95% CI: 1.2, 1.3) and 8.1 times higher among adults living in households where someone did not smoke inside on most days compared with households where someone did (95% CI: 6.6, 10). Similar patterns also were observed when all the predictors were examined in relation to geometric mean cotinine [see Supplemental Material, Table 1 (http://dx.doi.org/10.1289/ehp.1103680)].

**Table 1:** Factors associated with undetectable cotinine levels in nonsmoking adults and impacts of smokefree legislation, Health Survey for England, 1998–2008.

Sample (*n*)	Percent with undetectable cotinine	Unadjusted OR,*a*univariate regression		Adjusted OR,*a*multivariate regression*b*	
		Predictor variable	Levels in predictor variable	Estimate (95% CI)		Estimate (95% CI)	
Smokefree legislation		Before 1 July 2007									—	
		After 1 July 2007									1.52	(1.29, 1.79)**
Age (years)		16–29		4,352		20.7		—			—	
		30–44		8,087		27.7		1.47	(1.33, 1.62)**		1.56	(1.41, 1.73)**
		45–59		8,031		29.2		1.58	(1.44, 1.75)**		1.76	(1.59, 1.95)**
		≥ 60		9,815		33.5		1.93	(1.75, 2.13)**		2.21	(1.99, 2.46)**
Sex		Male		13,570		27.1		—			—	
		Female		16,715		30.0		1.15	(1.10, 1.21)**		1.23	(1.17, 1.30)**
Social class of head of household		I and II (professional, managerial, and technical)		13,546		32.9		—			—	
		III (skilled nonmanual and manual)		11,333		26.1		0.72	(0.68, 0.77)**		0.81	(0.76, 0.87)**
		IV and V (semiskilled and unskilled manual)		4,719		23.3		0.62	(0.57, 0.68)**		0.71	(0.65, 0.79)**
Education level of individual		Higher education qualification		9,518		32.6		—			—	
		School level (or other) qualifications		13,183		26.9		0.76	(0.72, 0.81)**		0.92	(0.86, 0.98)*
		No qualification		7,570		27.0		0.77	(0.71, 0.82)**		0.81	(0.74, 0.89)**
Ethnicity		White*c*		28,225		28.7		—			—	
		Black or Asian		1,638		28.7		1.00	(0.88, 1.15)		0.94	(0.81, 1.09)
Someone smokes most days inside the home?		Yes*c*		2,858		4.4		—			—	
		No		27,420		31.3		9.90	(8.09, 12.1)**		8.11	(6.57, 10.0)**
—, indicates baseline category. **a**Compares the ratio of the odds between a category and the baseline category. These estimates were derived by exponentiating the regression coefficients from the regression model. Regression results rounded to two decimal places.**b**Model includes a linear and quadratic term for time. **c**Includes qualifications obtained outside the United Kingdom, Nursery Nurse Examination Board, and Clerical and Commercial qualification. *p < 0.05 and **p < 0.01 indicates a significant difference from the baseline category.												

**Figure 1:**
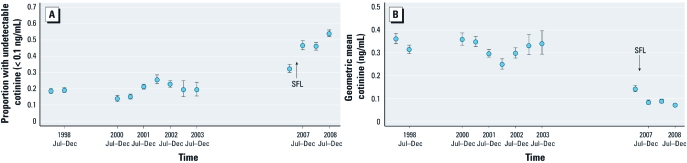
Trends in SHS exposure in nonsmoking adults in England from 1998 to 2008 in 6-month time periods using the proportion with undetectable cotinine (*A*), and geometric mean cotinine (*B*). Error bars indicate 95% CIs. SFL (smokefree legislation) shows the date when legislation was implemented.

*Impact of legislation.* Overall impact of the smokefree legislation. The percentage of nonsmokers with undetectable cotinine increased considerably over time from 19% (95% CI: 17%, 20%) in the first half of 1998 to 54% (95% CI: 52%, 56%) in the second half of 2008 with a clear increase seen between the first and second 6-month periods of 2007 (Figure 1A). Similarly, geometric mean cotinine declined from 0.36 ng/ml (95% CI: 0.34, 0.39) in the first half of 1998 to 0.071 ng/ml (95% CI: 0.066, 0.077) in the second half of 2008 (Figure 1B).

Assuming a quadratic prelegislative trend in the log odds of exposure and adjusting for the sociodemiographic predictors, the odds of having undetectable cotinine were 1.5 (95% CI: 1.3, 1.8) times higher after the smokefree legislation was introduced than before it was introduced (Table 1).

Evidence of a significant impact of the legislation was further supported by our second analysis that showed the OR for undetectable cotinine between the second and first halves of 2007 (postlegislation vs. prelegislation) was 1.8 (95% CI: 1.5, 2.2), significantly higher than that for all other 6-month comparisons in the 9 years preceding legislation (their ORs ranged from 0.88 to 1.3; *p* < 0.05 in all comparisons), with the exception of the OR comparing the second and first half of 2001 (OR = 1.5, *p* = .08) [see Supplemental Material, Figure 1 (http://dx.doi.org/10.1289/ehp.1103680)].

Significant reductions in exposure following smokefree legislation also were observed using both analyses when geometric mean cotinine was used as the outcome variable, with the first analysis identifying a 27% (95% CI: 17%, 36%) fall in geometric mean cotinine [see Supplemental Material, Table 1 (http://dx.doi.org/10.1289/ehp.1103680)] and the second analysis showing that the percentage fall, derived from the ratio of geometric mean cotinine, between the second half (1 July through 31 December) and the first half (1 January through 30 June) of 2007 (postlegislation vs. prelegislation) was 38% (95% CI: 28%, 46%), significantly greater than all other 6-month comparisons in the 9 years preceding legislation (percentage change ranged from a fall of 13% to an increase of 118%; *p* < 0.05 in all comparisons) [see Supplemental Material, Figure 1 (http://dx.doi.org/10.1289/ehp.1103680)].

**Figure 2:**
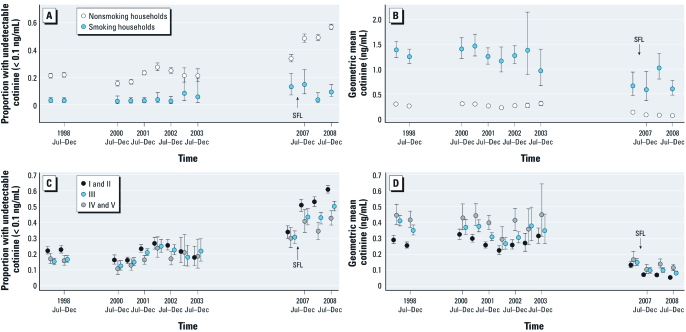
Trends in SHS exposure among nonsmoking adults in England from 1998 to 2008 in 6-month time periods by household smoking status (*A* and *B*) and social class of head of household (*C* and *D*) using proportion with undetectable cotinine (*A* and *C*), geometric mean cotinine (*B* and *D*). Error bars indicate 95% CIs. SFL (smokefree legislation) indicates when the legislation was implemented.

Variation in the estimated impact of the smokefree legislation by household smoking status and social class. The impacts of legislation appeared to vary by population subgroup (Table 2, Figure 2A–D). Significant impacts were observed only among those living in homes where there was no smoking inside on most days and among those from social classes I to III. For those living in homes where there was no smoking inside on most days, the odds of having undetectable cotinine were 1.6 (95% CI: 1.3, 1.9) times higher after the legislation was implemented and geometric mean cotinine fell by 31% (95% CI: 21%, 39%) after adjusting for underlying trends and potential confounders. The odds of having undetectable cotinine were 1.8 (95% CI: 1.4, 2.3) times higher among those in social classes I and II and 1.5 (95% CI: 1.1, 1.9) times higher among those in social classes III after the legislation, whereas geometric mean cotinine levels fell by 37% (95% CI: 24%, 48%) and 23% (95% CI: 6%, 37%) respectively. By contrast, no significant impact was seen in adults living in households where someone smoked inside on most days or in social classes IV and V when measured using either the OR of undetectable cotinine [0.38 (95% CI: 0.12, 1.2) and 1.00 (95% CI: 0.64, 1.6) respectively], or multiplicative change in geometric cotinine [1.5 (95% CI: 0.89, 2.5) and 1.0 (95% CI: 0.7, 1.4) respectively].

**Table 2:** Estimated impacts (95% CI) of smokefree legislation on SHS exposure among nonsmoking adults by social class and by whether smoking occurred inside the home on most days.

Predictor variable*a*	Levels in predictor variable	OR for undetectable cotinine*b*		Multiplicative change in geometric mean cotinine*c*	
Social class (head of household)		I and II (professional, managerial and technical)		1.76	(1.38, 2.25)*		0.63	(0.52, 0.76)*
		III (skilled nonmanual and manual)		1.48	(1.14, 1.92)*		0.77	(0.63, 0.94)*
		IV and V (semi-skilled and unskilled manual)		1.00	(0.64, 1.56)^#^		1.01	(0.70, 1.44)^#^
Someone smokes most days inside the home?		Yes		0.38	(0.12, 1.16)		1.48	(0.89, 2.47)
		No		1.58	(1.34, 1.86)*^#^		0.69	(0.61, 0.79)*^#^
**a**Results for social class based on multivariate models with predictors (i.e. smokefree legislation, age, sex, social class, education, ethnicity, whether someone smokes most days inside the home, and a linear and quadratic term for time) and two interactions—the first between social class and the linear and quadratic terms for time and the second between social class and the binary indicator of smokefree legislation. Results for household smoking status are based on the same models but with household smoking status replacing social class in the interaction terms. Results rounded to two decimal places. **b**Ratio in odds of undetectable cotinine after the legislation was introduced on 1 July 2007 compared with before it was introduced, derived from the smokefree legislation predictor and its interaction term. **c**Ratio of geometric mean cotinine after the legislation was introduced on 1 July 2007 compared with before its introduction, derived from the smokefree legislation predictor and its interaction term. *Significantly different (*p* < 0.05) from 1 (no change). ^#^Significantly different (*p* < 0.05) from baseline category (either social class I and II or answering “yes” to whether someone smoked most days inside the home).								

These patterns were largely confirmed in the second analysis comparing the odds across 6-month periods. For those living in homes where there was smoking inside on most days and from social classes III, IV, and V, there was no evidence that the OR of undetectable cotinine or the decrease in geometric mean cotinine between the first and second halves of 2007 were significantly greater than in other 6-month comparisons in the 9 years preceding legislation. By contrast, for those living in homes with no smoking inside the home or for those in social classes I and II, the OR of undetectable cotinine between the first half and the second half of 2007, 1.9 (95% CI: 1.6, 2.2) and 2.0 (95% CI: 1.6, 2.6) respectively, were significantly higher than were those for all 6-month comparisons in the 9 years preceding legislation (range, 0.84–1.3 and 0.78–1.2 respectively; *p* < 0.05 in all comparisons) with the exception of the OR between the first half and the second half of 2001 (1.5 and 1.6 respectively, *p* > 0.05). For those living in homes with no smoking inside or for those in social classes I and II, the percentage fall in geometric mean cotinine between the first and the second half of 2007 was 39% (95% CI: 30%, 47%) and 44% (95% CI: 32%, 54%), respectively; these percentages were significantly greater than all 6-month comparisons in the 9 years preceding legislation (percentage change ranged from a fall of 14% to a rise of 118% and a fall of 13% to rise of 117%, respectively; *p* < 0.05 in all comparisons; data not shown).

## Discussion

Levels of SHS exposure among nonsmoking adults declined significantly after smokefree legislation was introduced in England. After adjusting for prelegislative trends and other factors that may influence exposure, we found that the odds of having undetectable cotinine were 1.5 times higher after the legislation and that geometric mean cotinine levels fell by 27%. This reduction in SHS exposure among nonsmoking adults is consistent with that observed after the introduction of smoking bans in New York (Bauer et al. 2007) and Scotland (Haw and Gruer 2007) (geometric mean saliva cotinine levels declining by 47% and 39% respectively). The smaller estimated impact observed in England may be partly explained by our adjustment for the prelegislative trend, an issue not considered in the other studies, and by the lower levels of prelegislative exposure [certainly compared with Scotland where the geometric mean cotinine concentration for the 7-month period preceding their smokefree legislation was 0.43 ng/ml (Haw and Gruer 2007) compared with 0.14 ng/ml in our study].

The major strength of this study is that it was based on a large and representative survey of the general population using cotinine as a biomarker of SHS exposure. Although cotinine is a metabolite of nicotine, and it is not the nicotine itself that causes harm, studies have shown that cotinine concentrations correlate with those of carcinogenic compounds in cigarette smoke (Hecht et al. 2001) and are associated with elevated risks of cardiovascular and respiratory disease (Chan-Yeung and Dimich-Ward 2003; Whincup et al. 2004). Our study is one of only a few that has examined the impact of smokefree legislation on population level exposure to secondhand smoke, and it has advantages over other studies both in using a variety of outcome measures and methods and in taking into account the underlying downward trend in exposure observed between 1998 and 2008.

The main weakness of this study is the absence of cotinine data from 2004 to 2006 and the resulting difficulty of specifying the trend in exposure during this period. Incorrect trend specification might bias the estimated impact of smokefree legislation. However, we considered a number of trend options and, using the data available, we judged the quadratic trend to be the most appropriate. Furthermore, to avoid making any assumption about trend (and thus to overcome this problem) we performed a supplementary analysis in which the magnitude of the change between the 6 months before the legislation being implemented and the 6 months postlegislation was compared with that change observed in any other 6-month comparison, and our conclusions were the same. A further limitation is the effect that the timing of the saliva sample collection might have had on cotinine levels given evidence that cotinine levels are lower in the morning than in the evening (Jarvis et al. 1984). In the Health Survey for England, nurse visits were relatively more common after 1700 hours for those in professional and managerial households (Mindell et al. 2011). Thus differences in cotinine levels between professional and managerial households compared with lower-SES households may be attributable in part to the timing of the saliva sample collection. However, if the bias is consistent over time, it cannot account for the differences in trend between these groups. Finally, it is important not to interpret the reported OR as prevalence rate ratios, because ORs will always be further away from 1 (the null value) than will prevalence rate ratios (except when the latter is equal to 1), and the discrepancy is more pronounced when the outcome is relatively common, as is the proportion with undetectable cotinine (Zocchetti et al.1997).

This study also documents marked declines in SHS exposure among nonsmoking adults between 1998 and 2008 (the proportion with undetectable cotinine was 2.9 times higher and geometric mean cotinine fell by 80%). This is in line with the decline of 59% in geometric mean cotinine observed in children over a similar period (1996–2006) (Sims et al. 2010b). Such trends likely reflect the strengthening of tobacco control strategies during this period resulting in declining smoking rates [from 27% in 1998 (Department of Health 2000) to 21% in 2008 (NHS Information Centre 2010b)], changing knowledge (Evans 2010) and a growth in voluntary home-smoking restrictions (Jarvis et al. 2009) as well as increasing restrictions on smoking in public places implemented in advance of the legislation. Salivary cotinine concentrations declined both among those living in homes where no smoking occurs inside, from a geometric mean of 0.29 (95% CI: 0.27, 0.31) in the first 6 months of 1998 to 0.062 (95% CI: 0.058, 0.067) in the last 6 months of 2008 and among those living in homes in which smoking occurs inside on most days, from a geometric mean of 1.4 (95% CI: 1.2, 1.6) in the first 6 months of 1998 to 0.60 (95% CI: 0.47, 0.77) in the last 6 months of 2008. While the former must reflect changes in exposure outside the home, the latter could reflect reductions in exposure both outside and inside the home (if those smoking inside the home reduced their consumption or made attempts to smoke elsewhere). Nevertheless, salivary cotinine concentrations remained much higher among adults exposed at home and somewhat higher among lower-SES groups (Figure 2).

Living in a home where smoking is allowed was identified as an important determinant of exposure. Other factors were also important and remained so even after controlling for household smoking status. Consistent with other studies (Ellis et al. 2009; Jarvis et al. 2001; Pirkle et al. 2006), our findings showed that men and persons in the youngest age groups were most exposed, as were those from lower-SES groups.

Although the impact of legislation on exposure among nonsmokers varied by household smoking status and social class, there was no significant difference by age group (results not shown). It is noteworthy that smokefree legislation had little impact upon those who are most exposed to SHS: nonsmokers living in households where smoking occurs inside on most days and those from lower social classes. A similar pattern, of a significant reduction in exposure among nonsmoking adults from nonsmoking but not smoking households, was observed in Scotland (Haw and Gruer 2007), although another study reported that smoking bans in the United States had no effect on exposure among nonsmoking adults regardless of household smoking status (Adda and Cornaglia 2010). It is likely that for nonsmokers living in households where smoking occurs inside, home exposure is such a significant component of total SHS exposure that smokefree legislation cannot materially reduce their exposure. Similarly it appears that for those in lower socioeconomic households, exposure in public and work settings must have been a smaller component of total exposure. Although there is a clear increase in the number of smokefree homes in England with the proportion of nonsmokers living in households that are largely smokefree increasing over time from 87% in 1998 to 94% in 2008, our findings underline the importance of finding further ways to reduce exposure among the most exposed groups, notably those exposed at home and those from lower-SES groups and the importance of continuing to monitor exposure given that data presented in Figure 2 suggest that exposure in these groups may have leveled off postlegislation. Importantly, however, there was no significant increase in exposure among any groups.

## Conclusion

Smokefree legislation in England led to significant reductions in population exposure to SHS. These reductions were additional to already declining exposures, which in turn, likely reflects the success of tobacco control policies implemented over the period examined. Although secular declines in exposure were seen among all population groups, the study was unable to detect significant reductions in exposure following the legislation in population subgroups with the greatest exposure (those living in homes where smoking occurs inside on most days and those in lower-SES households). Although we were unable to formally examine trends in inequalities due to the periods of missing data, our data suggest that absolute inequalities in exposure declined up to 1 July 2007 (when measured using geometric mean cotinine), but subsequently increased after smokefree legislation was implemented, albeit affecting an ever smaller proportion of the population. This highlights the need both for further follow-up, requiring regular population-based cotinine surveillance data, and for further efforts to reduce SHS exposure to benefit those who remain most exposed.

## Supplemental Material

(143 KB) PDF
