# Supplementary material for: Did Smokefree Legislation in England Reduce Exposure to Secondhand Smoke among Nonsmoking Adults? Cotinine Analysis from the Health Survey for England
Source: Environ Health Perspect. 2011 Dec 12;120(3):425–30. doi: 10.1289/ehp.1103680 (PMC3295343; doi:10.1289/ehp.1103680)
Supplement: (143 KB) PDF [file ehp.1103680.s001.pdf]

SUPPLEMENTAL MATERIAL

Did Smokefree Legislation in England Reduce Exposure to Secondhand Smoke among Non-Smoking adults? Cotinine Analysis from the Health Survey for England

Michelle Sims, Jennifer S Mindell, Martin J Jarvis, Colin Feyerabend, Heather Wardle, Anna Gilmore

Table of Contents

	Pages
Creating the dummy variables for a logistic regression model to compare secondhand smoke exposure between two time periods.	2-6
Supplemental Material, Table 1	7
Supplemental Material, Figure 1	8

Creating the dummy variables for a logistic regression model to compare secondhand smoke exposure between two time periods.

In this section we describe how the dummy variables in the logistic regression model were coded so that the odds of having undetectable cotinine in a six month period with the odds in the previous six month period could be compared. A similar approach was also taken to compare geometric mean cotinine levels between a six month period and a preceding one.

1. Defining the dummy variable

Let Z equal the date that the nurse visited the respondent and p represent the proportion of non-smoking adults with undetectable cotinine. We define 12 dummy variables as follows:

$X_1 = 1 \text{ if Z} \geq \text{July 1998, 0 otherwise}$
$X_2 = 4 \text{ if Z} \geq \text{July 2000, 0 otherwise}$
$X_3 = 1 \text{ if Z} \geq \text{Jan 2001, 0 otherwise}$
$X_4 = 1 \text{ if Z} \geq \text{July 2001, 0 otherwise}$
$X_5 = 1 \text{ if Z} \geq \text{Jan 2002, 0 otherwise}$
$X_6 = 1 \text{ if Z} \geq \text{July 2002, 0 otherwise}$
$X_7 = 1 \text{ if Z} \geq \text{Jan 2003, 0 otherwise}$
$X_8 = 1 \text{ if Z} \geq \text{July 2003, 0 otherwise}$
$X_9 = 7 \text{ if Z} \geq \text{Jan 2007, 0 otherwise}$
$X_{10} = 1 \text{ if Z} \geq \text{July 2007, 0 otherwise}$
$X_{11} = 1 \text{ if Z} \geq \text{Jan 2008, 0 otherwise}$
$X_{12} = 1 \text{ if Z} \geq \text{July 2008, 0 otherwise}$

A logistic regression model to explore odds ratios between a six month period and the preceding one is given by:

$$\log\left(\frac{p}{1-p}\right) = \beta_1 X_1 + \beta_2 X_2 + \beta_3 X_3 + \beta_4 X_4 + \ldots + \beta_{10} X_{10} + \beta_{11} X_{11} + \beta_{12} X_{12} \quad (1)$$

In our analysis, equation (1) also includes an intercept and the predictors listed in Table 1, but without the linear and quadratic terms for *time* and the binary smokefree legislation predictor. The exponential of a regression coefficient β associated with a dummy variable coded 0 or 1 represents the odds ratio between a six month period and the preceding one. For example, $\exp(\beta_1)$ is the odds ratio between July-December 1998 and January-June 1998.

During periods when no cotinine data were collected, we assume that the odds ratios between a six month period and the preceding one remain constant and we can estimate this constant odds ratio by recoding the 1 in the dummy variable associated with this time period with a value equal to the number of missing six month comparisons. For example, no cotinine data were collected for the period 1999 and January to June 2000. If this data had been available, we would have calculated four odds ratios between July 1998 and July 2000 (i.e. four comparisons of a six month period to the preceding one) and we therefore use the value 4 in X_2 instead of 1. To demonstrate how the constant odds ratio is derived, we define the odds of having undetectable cotinine for each 6 month period from July 1998 to December 2000 as:

$$\begin{aligned}
O_1 &= \text{July - December 1998} \\
O_2 &= \text{January - June 1999} \\
O_3 &= \text{July - December 1999} \\
O_4 &= \text{January - June 2000} \\
O_5 &= \text{July - December 2000}
\end{aligned}$$

From equation (1), the odds ratio between July-December 2000 (O_5) and July-December 1998 (O_1) is calculated as $\exp(\beta_2)^4$. If data for 1999 and January-June 2000 had been available, we could also have obtained this odds ratio by multiplying together the four odds ratios that compare a 6 month period to the preceding one between these dates, i.e.

$$\frac{O_5}{O_1} = \frac{O_2}{O_1} \times \frac{O_3}{O_2} \times \frac{O_4}{O_3} \times \frac{O_5}{O_4}.$$

As no data is available, we assume the odds ratios for the six month comparisons is a constant value, *C*, and then

$$C = \frac{O_2}{O_1} = \frac{O_3}{O_2} = \frac{O_4}{O_3} = \frac{O_5}{O_4}$$

and

$$\frac{O_5}{O_1} = C^4 = \exp(\beta_2)^4.$$

With this assumption in place, $\exp(\beta_2)$ equals C, the constant odds ratio.

A similar approach was used to estimate odds ratios for the period 2004 to 2006 when cotinine data were again not collected.

2. Examples of six month comparisons

Example (i): Comparing July-December 2001 with January-June 2001

The odds of having undetectable cotinine in July-December 2001 is:

$$O_1 = \frac{p}{1-p} = \exp(\beta_1 + 4\beta_2 + \beta_3 + \beta_4)$$

The odds of having undetectable cotinine in January-June 2001 is:

$$O_2 = \frac{p}{1-p} = \exp(\beta_1 + 4\beta_2 + \beta_3)$$

Therefore the odds ratio between these two periods can be calculated as:

$$\frac{O_1}{O_2} = \exp(\beta_4)$$

Example (ii): Comparing a six month period with the preceding one between July 2003 and June 2007.

The odds of having undetectable cotinine in January-June 2007 is:

$$O_1 = \frac{p}{1-p} = \exp(\beta_1 + 4\beta_2 + \beta_3 + \beta_4 + \ldots + \beta_8 + 7\beta_9)$$

The odds of having undetectable cotinine in July-December 2003 is:

$$O_2 = \frac{p}{1-p} = \exp(\beta_1 + 4\beta_2 + \beta_3 + \beta_4 + \ldots + \beta_8)$$

Therefore the odds ratio between these two periods is given by:

$$\frac{O_1}{O_2} = \exp(7\beta_9) = \exp(\beta_9)^7$$

If data for 2004-2006 had been available, we would have calculated seven odds ratios between July 2003 and June 2007 (i.e. seven comparisons of a six month period to the preceding one). As this data is not available, we let $\exp(\beta_9)$ represent the odds ratio between a six month period and the previous six month period, and assume it remains constant between July 2003 and June 2007.

3. Comparing two odds ratios

In our paper, we investigated whether the odds ratio between the second and first half of 2007 (i.e. a comparison of the six months post- and pre-legislation) was significantly higher than other six month comparisons between 1998 and 2008. In this section, we illustrate how to test for a significant difference between this and another odds ratio using the ratio of odds between the second and first half of 2001 as the comparison odds ratio.

We assume that the odds ratio between the second and first half of 2001 is $\exp(\beta_4)$, as was described in Example (i). We then set the odds ratio between the second and first half of 2007 to equal $\exp(\beta_4)$ multiplied by a factor N. We can test whether N is significantly different from 1, i.e. whether there is a difference in magnitude between the two odds ratios, by fitting the logistic regression model defined in equation (1) except X_4 is replaced with a new variable X_{410}:

$$X_{410} = 0 \text{ if Z} < \text{July 2001}$$
$$X_{410} = 1 \text{ if Z} \geq \text{July 2001 and Z} < \text{July 2007}$$
$$X_{410} = 2 \text{ if Z} \geq \text{July 2007}$$

The odds of having undetectable cotinine in July-December 2007 is then:

$$O_1 = \frac{p}{1-p} = \exp(\beta_1 + 4\beta_2 + \beta_3 + 2\beta_4 + ... + 7\beta_9 + \beta_{10})$$

The odds of having undetectable cotinine in January-June 2007 is:

$$O_2 = \frac{p}{1-p} = \exp(\beta_1 + 4\beta_2 + \beta_3 + \beta_4 + ... + 7\beta_9)$$

Therefore the odds ratio between the second and first half of 2007 is given by:

$$\frac{O_1}{O_2} = \exp(\beta_4)\exp(\beta_{10}) \qquad (2)$$

N is equal to $\exp(\beta_{10})$ in equation (2) and we can therefore test whether the magnitude of the two odds ratios are different by testing whether β_{10} is significantly different from zero.

Supplemental Material, Table 1. Factors associated with geometric mean cotinine levels in non-smoking adults and impacts of smokefree legislation (Health Survey for England data 1998-2008)

Predictor variable	Levels in predictor variable	Sample size (N)	Observed geometric mean	Multiplicative change[a] Univariate regression		Multiplicative change[a] Multivariate regression[b]	
				Estimate	(95% CI)	Estimate	(95% CI)
Smokefree legislation	before 1st July 2007[c]					-	-
	after 1st July 2007					0.73*	(0.64, 0.83)
Age	16-29[c]	4352	0.30	-	-	-	-
	30-44	8087	0.21	0.71*	(0.66, 0.76)	0.75*	(0.71, 0.80)
	45-59	8031	0.20	0.68*	(0.63, 0.73)	0.69*	(0.65, 0.73)
	60+	9815	0.17	0.56*	(0.52, 0.61)	0.58*	(0.54, 0.62)
Gender	male[c]	13570	0.23	-	-	-	-
	female	16715	0.19	0.83*	(0.80, 0.86)	0.80*	(0.77, 0.82)
Social class of the head of household	I and II[c] (professional, managerial and technical)	13546	0.17	-	-	-	-
	III (skilled non-manual and manual)	11333	0.24	1.40*	(1.33, 1.47)	1.17*	(1.13, 1.22)
	IV and V (semi-skilled and unskilled manual)	4719	0.28	1.68*	(1.58, 1.80)	1.32*	(1.24, 1.39)
Education	higher education qualification[c]	9518	0.17	-	-	-	-
	school level (or other) qualifications[d]	13183	0.23	1.36*	(1.30, 1.43)	1.11*	(1.06, 1.16)
	no qualification	7570	0.24	1.43*	(1.35, 1.51)	1.26*	(1.20, 1.33)
Ethnicity	white[c]	28225	0.21	-	-	-	-
	black or Asian	1638	0.19	0.92	(0.83, 1.01)	0.99	(0.90, 1.09)
Someone smokes most days inside the home?	yes[c]	2858	1.15	-	-	-	-
	no	27420	0.17	0.15*	(0.14, 0.16)	0.20*	(0.19, 0.21)

[a] It describes the ratio of geometric mean cotinine for a category relative to the geometric mean cotinine of the baseline category. These were derived by exponentiating the regression coefficients from the regression model. Results rounded to two decimal places.
[b] This model also includes a linear and quadratic term for time.
[c] Baseline category
[d] Also includes qualifications obtained outside of UK, Nursery Nurse Examination Board, Clerical and Commercial qualifications
*indicates a significant difference (P<0.01) from the baseline category

Supplemental Material, Figure 1. Comparison of secondhand smoke exposure in non-smoking adults in England in a six month period compared with the previous six month period using: a) odds ratio of the proportion with undetectable cotinine[a], b) ratio of geometric mean cotinine. Error bars indicate 95% confidence intervals. SFL shows the odds ratio comparing the six months prior to the legislation being implemented with the six months post-legislation.

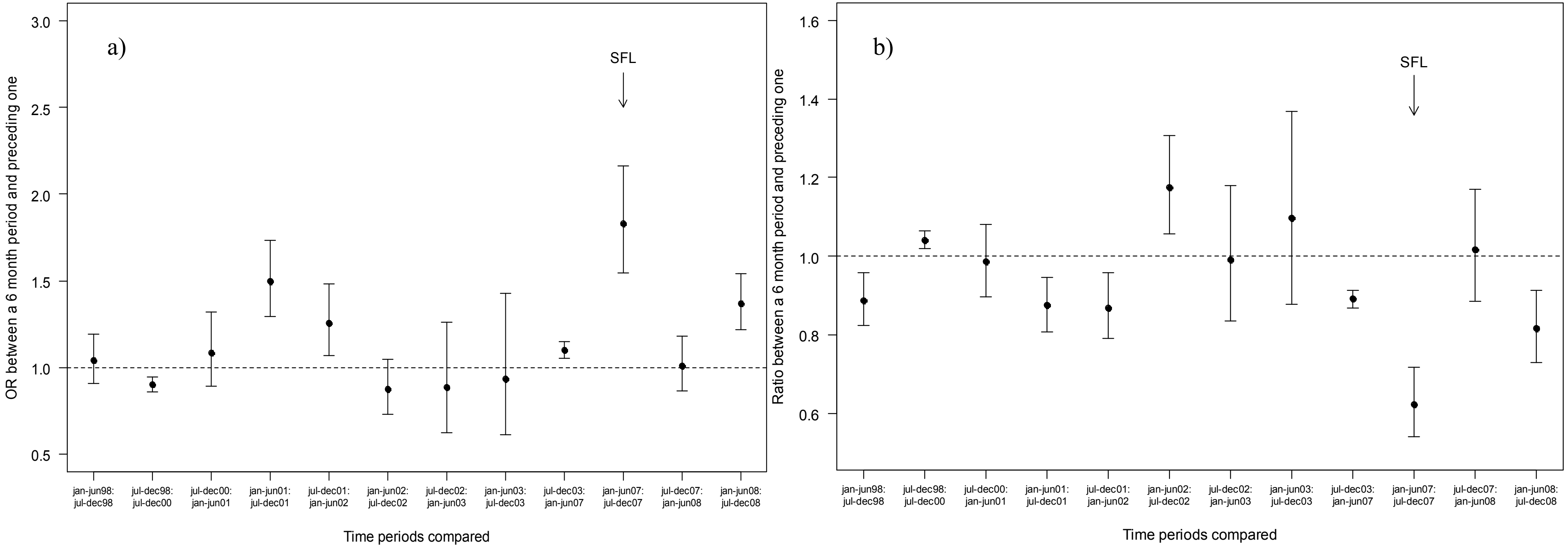

[a] for example: jan-jun03:jul-dec03 represents the odds of having undetectable cotinine in July to December 2003 compared with January to June 2003. jul-dec03:jan-jun07 represents the ratio odds of having undetectable cotinine in a six month period compared with the previous six month period between July 2003 and June 2007 (i.e. we assume a constant odds ratio for each six month comparison during this time period).
